# Study of hydronephrosis and renal function reversibility on different development stages of rats that underwent unilateral ureteral obstruction, followed by its release through pyelostomy

**DOI:** 10.1590/acb408325

**Published:** 2025-11-10

**Authors:** Ana Cristina Aoun Tannuri, Victor Kenji Utino Kitahara, Alexandre Amatruda Marum, Bruno Mola, Daniel Felipe Marcelino Souza, Lucas Menon Rodrigues, Vitor Ribeiro Paes, Katia Ramos Moreira Leite, Suellen Serafini, Josiane de Oliveira Gonçalves, Uenis Tannuri

**Affiliations:** 1 Universidade de São Paulo – Faculdade de Medicina– São Paulo (SP) – Brazil.; 2 Universidade de São Paulo – Faculdade de Medicina – Laboratório de Investigação Médica em Cirurgia Pediátrica – São Paulo (SP) – Brazil.

**Keywords:** Kidney Diseases, Ureteral Obstruction, Hydronephrosis

## Abstract

**Purpose:**

To verify the differences in the reversibility of renal function and damage in rats of different ages with unilateral ureteral obstruction (UUO).

**Methods:**

Sixty rats were divided into three different groups, newborns, young, and adults. Twenty-one were sham operated. After UUO, the animals were subdivided into four groups: obstruction (two weeks and sacrifice O2); UUO two weeks, obstruction release for two weeks and sacrifice (R2); UUO for four weeks and sacrifice (O4); and UUO for four weeks, obstruction release for two weeks and sacrifice (R4). Urine was collected for creatinine, urea, total protein, and albumin dosages, and kidneys for morphological studies.

**Results:**

Younger rats presented more extensive destruction of renal parenchyma, with some remaining normal tissue healthier in comparison to older rats. The protein excretion by older rats was not different between the groups obstructed for different periods, albuminuria was progressively higher in rats obstructed for longer periods, and after relief of obstruction, there was no difference between newborn rats obstructed for different periods. The younger pyelostomized rats presented higher albumin excretion. Creatinine excretion was worsened in rats after obstruction.

**Conclusion:**

Shorter obstruction periods lead to better prognosis, and younger rats showed better recovery after relief of obstruction than older ones.

## Introduction

Obstructive nephropathy is a renal disorder that begins with hydrodynamic and hemodynamic responses, ultimately leading to cellular changes in all renal compartments, and, finally, to renal interstitial fibrosis and tubular atrophy. Although it causes progressive loss of renal function, its diagnosis is challenging because the condition most often exhibits minimal or even no symptoms and signs. However, once the obstruction is recognized, treatment is simple, making early diagnosis important for a good prognosis^1^.

In infants and children, congenital obstructive nephropathy is a major etiology of renal insufficiency, most frequently caused by the obstruction of the ureteropelvic junction, which occurs in about 1% of infants and children submitted to ultrasonographic examination^1^, accounting for 10–30% of cases of pediatric hydronephrosis^2^.

It is known that early treatment of obstructive nephropathy leads to better overall prognosis. We believe that, due to their greater plasticity, children and infants have a higher tolerance for hydronephrosis and can also recover better from the injury caused by obstruction, when compared with individuals at an older age. To confirm this hypothesis, we developed a model of complete unilateral ureteral obstruction (UUO) in rats followed by pyelostomy, as the form of obstruction relief. Although partial UUOs are more prevalent than complete obstructions in humans, we chose complete obstructions because their effects are more severe, and for being more accessible and cheaper to reproduce than partial obstructions^3^^,^ 4. We sought to confirm if there is any difference in the reversibility of renal function and damage in different durations of obstruction and development stages in rats.

## Methods

The animals were cared for following the criteria set forth in the Guide for Care and Use of Laboratory Animals from the National Academy of Sciences. The study protocol was reviewed and approved by the Animal Ethics Committee of the present study’s institution, protocol number 030-17.

### Groups

We compared three different age groups of Wistar rats: newborns (N), which were 2-week-old rats; young (Y), which were 4-week-old rats; and adults (A), which were 8-week-old rats. Unlike humans, in whom nephrogenesis is complete before birth, only 10% of nephrons are formed at birth in rats, with the remainder developing in the first postnatal week of life^5^^–^ 7. Therefore, we chose these age groups to mimic infants and young children (both represented by newborns), teenagers (represented by young rats), and adults.

**Figure 1:**
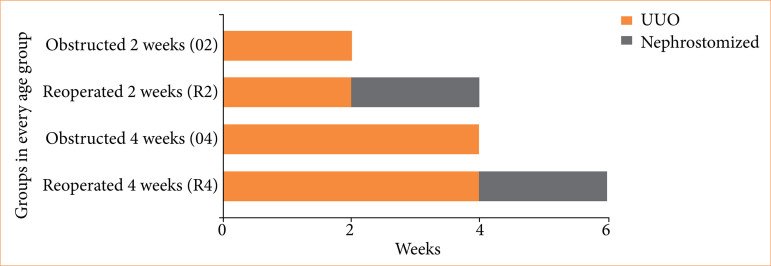
Scheme showing group division inside every age group studied. After all periods, rats were sacrificed for the collection of kidneys and urine.

Eighty-one rats of both sexes were distributed in sham-operated (21 rats) and operated groups (60 rats). At every age group, there were 20 rats subjected to UUO, then they were divided into four groups of five animals each. The first group was subjected to UUO for two weeks (O2) and sacrificed after that period. The second group was subjected to UUO for two weeks and then was released (R2) through pyelostomy for two more weeks and, after that, they were sacrificed. The third group of animals, like the first one, was subjected to UUO for four weeks (O4) and sacrificed after that period. The fourth and last group was subjected to UUO for four weeks, then released (R4) through pyelostomy for two more weeks and, after that, the animals were sacrificed (Fig. 1).

UUO: unilateral ureteral obstruction. Source: Elaborated by the authors.

Since there were a lot of groups, we created a code to identify each group. The code is formed by the age group of the rat followed by the operation group it is in (O2, R2, O4, R4). For example, a newborn rat from the group who was subjected to UUO for two weeks and sacrificed afterwards is identified as NO2.

The sham-operated rats were randomized in groups of three rats of 4, 6, and 8 weeks of age. They were subjected to midline abdominal incision and then a simple suture of skin. The choice of the rat age for the sham group is associated with their kidney development. We noticed that rats ranging from 8–14 weeks old had very similar kidneys. Therefore, we used the same sham group to compare all rats older than 8 weeks.

### Operations

The UUO was made through a midline abdominal incision followed by ligation and section of the left ureter with 8-0 prolene suture, on its distal third. Finally, the animal abdominal wall was closed utilizing 5-0 prolene sutures;

The relief of obstruction was made through a left flank incision right over the palpable left hydronephrotic kidney. Then the dilated pelvis was opened, and a pyelostomy was performed with eight separated stitches attaching the wall of the renal pelvis to the skin.

### Material collection

At the end of the experiment, the rats were sacrificed, and both kidneys were removed for studies.

There were three types of urine collection in this experiment.

Sham-operated rats remained 24 hours in a metabolic cage.

For the rats subjected to UUO without relief, the urine was collected by pelvic puncture from the hydronephrotic kidney.

For the rats subjected to UUO and relief thereafter, the urine was collected directly from the pyelostomy using collection bags for 24 hours, utilizing procedures according to previous publications^8^^,^ 9.

### Data analyses

#### Histological and histomorphometric analyses

Two experienced pathologists studied all 162 removed kidneys for histological qualitative analyses. Besides, the histomorphometric variables analyzed were the parenchyma thickness of both operated and contralateral kidneys. We also adopted a correction coefficient based on the average control data, so that the variable chosen would be more trustworthy. The variables calculated were parenchyma thickness and parenchyma corrected by control.

#### Biochemical analysis

Among all the 55 urine samples, 48 were successfully collected, and creatinine (Cr), urea (U), total protein (TP), and albumin (Alb) were dosed. Along with that, total protein/creatinine (TP/Cr) relations were calculated for further analysis^10^.

#### Statistical methods

The statistical analyses were made by using t-tests and Mann-Whitney tests. T-tests were used in groups with parametric data, whereas Mann-Whitney tests were used in non-parametric data. We compared all variables of every rat with its corresponding sham-operated rats (S4, S6, S8). Then, we compared the groups in the same age group (O2 with R2, O2 with O4, R2 with O4, R2 with R4, and O4 with R4), and the same groups from different age groups (NO2 with YO2 and with AO2, for example).

## Results

### Qualitative analysis

It was observed that, in all ages, the relieved kidneys had a superposition of acute and chronic inflammatory stages, associated with the opening of some of the renal tubules that collapsed in the kidneys of obstructed groups. The superposition of inflammatory states may be justified by the second surgical stress to the organ and by a possible process of remodeling in the organ after relief. After pyelostomy, comparisons between different age groups showed that the reopening of tubules in younger rats (newborns and young groups) had an aspect close to a healthy kidney’s parenchyma. In these animals, pyelostomy promoted the effect of preserving more portions of healthy-like tissue than the older age groups, despite the previous damage and atrophic findings on the rest of the kidney parenchyma.

The pattern of damage in adult groups was that of distension of the tubules with hyaline material, glomerular fibrosis, dystrophic calcification, and thickening of the tunica intima of the terminal arterioles.

**Figure 2:**
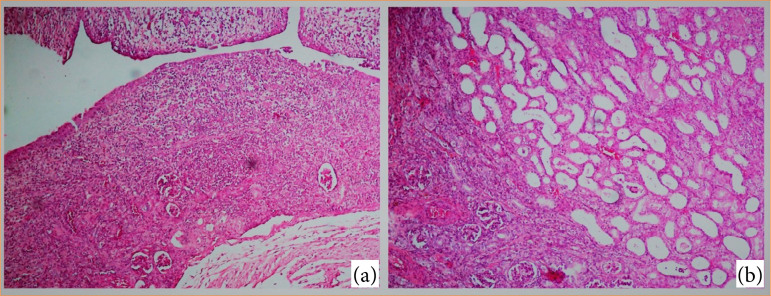
Histological aspects of the obstructed ureter kidney. **(a)** Acute inflammation, macrophage cells, and tubular atrophy. **(b)** Tubular swelling with signs of acute inflammation, tubular swelling, and fibrotic areas.

Source: Elaborated by the authors.

**Figure 3:**
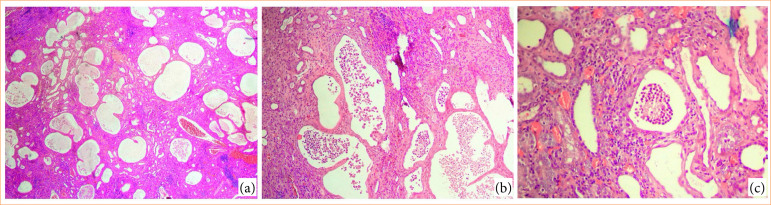
Additional histological aspects observed in the obstructed ureter kidney. **(a)** Significant dilation and expressive tortuosity of renal tubules. (**b** and **c**) Inflammation with presence of macrophage cells and debris formation of inflammatory cells into the tubules’ lumen.

Source: Elaborated by the authors.

Regarding the glomeruli, it was observed that, in all animals the ureteral obstruction promoted less intense lesions compared with the tubular damage. An important observation was that regarding glomeruli of obstructed kidneys for two weeks, in the newborn kidneys more glomeruli were preserved, whereas in the young kidneys there was a pattern of atrophic glomeruli, and in the adult kidneys there was a tendency for mostly fibrosed glomeruli (Figs. 2 and 3).

### Histomorphometry

#### Renal parenchyma thickness

In all studied groups, there was a statistical difference between the kidneys of obstructed rats and the sham-operated rats.

For the analysis of the renal parenchyma thickness between ages, as cited above, we utilized the measurements from sham-operated groups in the respective ages as correction parameters.

#### Obstructed kidneys

In newborns, the mean renal parenchyma thickness in NO2 was lower than S4 (*p* = 0.0179), and NBR2 was lower than S6 (p = 0.0179). The same difference was observed in NBO4 *versus* S6 (*p* = 0.0179), and NBR4 *versus* S8 (*p* = 0.0179).

In young animals, YR2 value was lower than S8 (*p* = 0.0357), S8 was thicker than YO4 (*p* = 0.0179), and YR4 was also thinner than S8 (p = 0.0179).

In adult animals, AO2 value was higher than AO4 (*p* = 0.0307), AR2 was thicker than AR4 (*p* = 0.0397), S8 was thicker than AO2 (*p* = 0.0286) and AR2 (*p* = 0.0179), and S8 was also thicker than AO4 (*p* = 0.0179).

**Figure 4:**
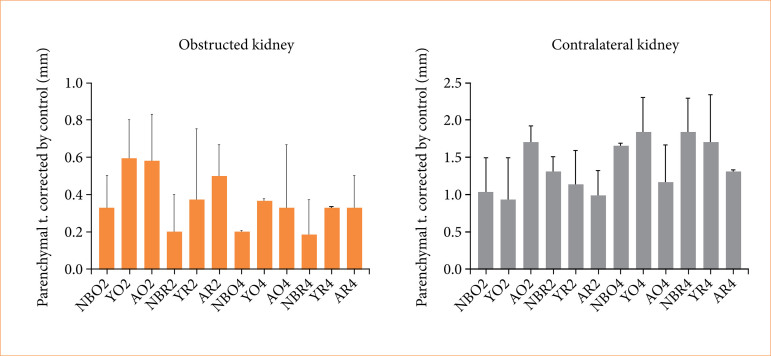
Histomorphometric data of obstructed and non-obstructed contralateral kidneys.

NB: newborn; Y: young; A: adult; O2: unilateral ureteral obstruction two weeks; R2: unilateral ureteral obstruction two weeks, obstruction release for two weeks and sacrifice; O4: unilateral ureteral obstruction for four weeks and sacrifice; R4: unilateral ureteral obstruction for four weeks, obstruction release for two weeks and sacrifice. Source: Elaborated by the authors.

Cross-age comparisons were also made, using corrections by sham-groups measurements: NBO2 was thinner than YO2 (*p* = 0.0222) and AO2 (*p* = 0.0069); NBR2 was also thinner than AR2 (*p* = 0.004); NBO4 value was thinner than AO4 (*p* = 0.0198); and finally, NBR4 was thinner than AR4 (*p* = 0.0159) (Fig. 4).

### Contralateral kidney

Analyzing groups solely by age, we highlight a few relevant comparisons. In newborns, the mean renal parenchyma thickness in R2 was lower than O4 (*p* = 0.0278), O4 was higher than S4 (*p* = 0.0357), and R4 was also higher than S6 (*p* = 0.0179).

In young rats, the mean renal parenchyma thickness in O4 was higher than O2 (*p* = 0.0317), and O4 was also higher than R2 (*p* = 0.0317). The same difference was observed in the comparison between R2 and R4, with R4 being thicker than R2 (*p* = 0.0317), and O4 mean renal parenchyma was thicker than S8 (*p* = 0.0179).

No statistical differences were observed in the comparisons of adult groups.

**Figure 5:**
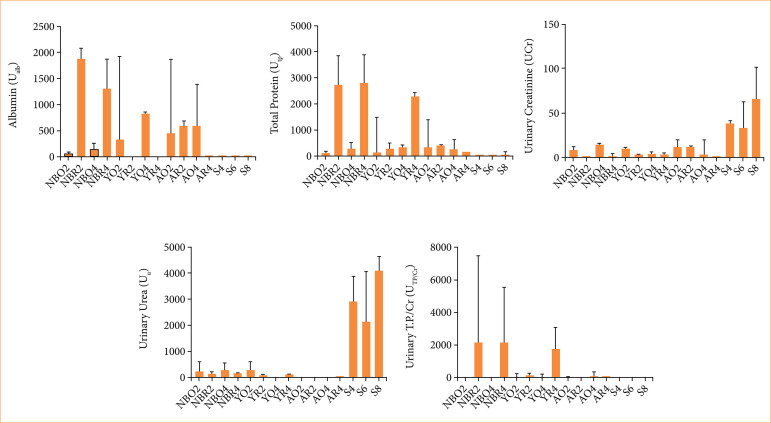
Results and comparisons of biochemical analyses of groups.

NB: newborn; Y: young; A: adult; O2: unilateral ureteral obstruction two weeks; R2: unilateral ureteral obstruction two weeks, obstruction release for two weeks and sacrifice; O4: unilateral ureteral obstruction for four weeks and sacrifice; R4: unilateral ureteral obstruction for four weeks, obstruction release for two weeks and sacrifice. Source: Elaborated by the authors.

Cross-age comparisons were made using correction parameters by sham-group measurements and provided statistical differences in a few groups. NBO4 mean renal parenchyma thickness was higher than AO4 (*p* = 0.0278), NBR4 was higher than AR4 (*p* = 0.004), and YO4 was higher than AO4 (*p* = 0.0232). All histomorphometric data are condensed in Fig. 5.

### Biochemical analysis

It was observed that after the relief of the adult age group, a few rats did not produce urine for collection (one animal in AR2, and two in AR4). The problem was also noticed in group YR4, in which only four out of the five reoperated rats produced urine. However, urine collection was possible in all newborn animals.

In newborn rats, the median of albuminuria in O2 was lower than in O4 (*p* = 0.0167). When comparing O2 with R2, the latter group was much higher (*p* < 0.0001). Also, the O4 showed less albuminuria than the R2 and R4 groups (*p* < 0.0001 and *p* = 0.0004, respectively). The albuminuria of R2 and O4 groups were both higher than S6 (R2 *versus* S6, *p* = 0.0003 and *p* = 0.0064), and R4 was higher than S8 (*p* = 0.0006). In young and adult rats, the difference was not as relevant. In young rats, R2 albuminuria was lower than O4 (*p* = 0.0005), and O4 level was higher than S6 (*p* = 0.0005). In the adult groups, O4 presented higher albuminuria than S6 group (*p* = 0.0333).

Looking at the cross-age comparisons, it was observed that NBR2 was higher than YR2 (*p* = 0.0003), NBO4 lower than YO4 (*p* = 0.0004), NBO4 was lower than AO4 (*p* = 0.0396), and NBR4 was higher than YR4 (*p* = 0.0222).

About TP, comparing the newborns, O2 *versus* R2 showed that the reoperated rats had a median higher than the obstructed rats (*p* = 0.0006). O4 *versus* R4 showed an increase after reoperation (*p* = 0.0006). Comparing the values of the operated groups with the sham groups, every comparison was relevant, with the operated groups showing more proteinuria than the sham groups: O2 *versus* S4 (*p* = 0.0303), R2 *versus* S6 (*p* = 0.0019), O4 *versus* S6 (*p* = 0.0088), and R4 *versus* S8 (*p* = 0.0061).

Analyzing cross-age values of proteinuria, we verified that NBR2 was higher than YR2 (*p* = 0.0032). NBO4 was lower than YO4 (*p* = 0.0024) and AO4 (*p* = 0.0433).

Regardind Cr, in newborns, we verified that only R2 *versus* R4 did not show relevance. O2 had lower Cr excretion than O4 (*p* = 0.0063) but had higher creatinuria than R2 (*p* = 0.0027). O4 had higher excretion than R4 (*p* < 0.0001). The operated groups presented decreased Cr excretion in comparison to the sham groups: O2 *versus* S4 (*p* = 0.0004), R2 *versus* S6 (*p* = 0.0034), O4 *versus* S6 (*p* = 0.0178), and R4 *versus* S8 (*p* = 0.0003).

In young rats, we verified that O2 presented higher creatinuria than R2 (*p* = 0.0175). The comparisons between operated groups with sham groups showed lower Cr excretion in operated animals: O2 *versus* S4 (*p* = 0.0223), R2 *versus* S6 (*p* = 0.0246), and O4 *versus* S6 (*p* = 0.0266).

In adult rats, we found differences only between two groups and their sham control groups, and like the differences in other age groups, the sham group presented higher Cr excretion, O2 *versus* S4 (*p* = 0.0189), and O4 *versus* S6 (*p* = 0.007).

Regarding the data between different age rats, NBO4 showed more creatinuria than YO4 (*p* = 0.0003) and AO4 (*p* = 0.0353).

About U firstly, analyzing the groups by age, we noticed that all newborn groups showed lower U excretion when compared with the sham group. O2 presented a lower U excretion than S4 (*p* = 0.0005). The R2 group also showed the same behavior when compared with its sham animals S6 (*p* = 0.006). The same was observed in O4 and S6 (*p* = 0.0079), and in R4 compared with its sham S8 (*p* < 0.0001).

In the young rat group, the O2 group presented higher U excretion than the O4 group (*p* = 0.0237). The O2 group also presented higher U excretion when compared with R2 (*p* = 0.0164). Comparing the data of young rats with their sham rats, operated rats showed lower U excretion: O2 *versus* S4 (*p* = 0.0224), R2 *versus* S6 (*p* = 0.0095), and O4 *versus* S6 (*p* = 0.0098).

In adult rats, O2 *versus* O4 did not show statistical relevance. However, the operated rats presented lower U excretion than their sham groups: O2 *versus* S4 (*p* = 0.0028), and O4 *versus* S6 (*p* = 0.0286).

Analyzing groups across different age groups, we noticed that NBO2 excreted more U than AO2 (*p* = 0.0192). Also, NBO4 excreted more U than AO4 (*p* = 0.0143). NBR4 also had higher U excretion than YR4 (*p* = 0.0444). The same pattern was observed in YO2 *versus* AO2 (p = 0.0025), and YO4 *versus* AO4 (*p* = 0.0286), with the younger rats presenting more urea excretion.

Studying the results from different age groups separately, in newborns the O2 group had a higher TP/Cr ratio than the O4 groups (*p* = 0.0318). Also, the newborn O4 group presented a lower ratio than R4 (*p* = 0.0191).

Analyzing the comparisons between age groups, we noticed that NBO4 had a lower ratio than YO4 (*p* = 0.0258).

All results and comparisons of biochemical analyses among groups are shown in Fig. 5.

## Discussion

Most of our understanding about the pathophysiology of kidney injury due to urinary tract obstruction comes from investigations performed in postnatal rat models. In previous publications, a partial UUO was performed, by embedding two-thirds of the left ureter in a psoas muscle tunnel. Our main criticism of this procedure is because it does not promote uniform obstruction in all operated animals. So, we decided to perform a complete obstruction and verify its reversibility in two periods of time, resubjected to severe partial UUO or sham operation according to a modification of the technique of Ulm and Miller, described by Wen et al.^11^.

### Histology and histomorphometry

As discussed in the histology section, after a post-renal injury, apparently younger organisms present a more damaged kidney than older rats, with more pronounced parenchymal loss, tubular atrophy, and tubular dilatation, but in both obstructed and unobstructed newborn rats the aspect of each remaining nephron was better than the one in older rats. When studying the renal function of rats, apparently younger rats show a better response to long-term obstruction than older rats, since newborn rats obstructed for two weeks show a similar protein and renal function loss compared to older rats with the same obstruction duration, but newborn rats obstructed for four weeks present less protein loss and better renal function than older rats submitted to the same injury. We believe that, even though the newer kidney suffers more from the obstructive injury histologically, thanks to its renal functional reserve and increased plasticity, it can adapt better to post-renal injury than older rats^12^. Probably the remaining nephrons can compensate better for the obstruction in developing animals than in older ones^13^.

So, we decided to use a urinary protein loss and urinary creatinine excretion ratio (Utp/Ucr) to understand those changes better. This ratio indicates renal injury, and it tends to increase after UUO and to be higher for a longer duration of obstruction^10^. After UUO, all rats tended to present an increase in Utp/Ucr when compared with sham rats. When comparing newborn rats, the Utp/Ucr ratio tended to be worse in a two-week UUO than in a four-week UUO, and for older rats, the ratio also tended to be worse the longer the obstruction. That may be justifiable because the younger kidney is probably more complacent than the older kidney, allowing the UUO to create more tissue destruction due to hydrostatic pressure than the older, more structurally mature kidney. That way the protein loss in the younger kidney is probably more important than that of the older ones, justifying this finding in the newborn group. When studying all rats, we learned that, although not much data had statistical differences, there was a tendency for similar ratios from a two-week obstruction in all age groups, but in a four-week obstruction, apparently older rats suffer more from the obstruction. After the relief of obstruction, we observed that younger rats tended to suffer a greater rise in both ratios. This finding may reflect an early phase of hyperperfusion or hyperfiltration of the unobstructed kidney, which tends to be more pronounced in younger organisms, and is likely associated with glomerular and tubular injury that has not yet been fully resolved.

It is known that ureteral obstruction leads to renal weight loss, and that complete obstruction stops the renal growth between 14 and 28 days of life^5^. The longer the obstruction time, the more intense the tubular damage (that also leads to parenchymal loss), and reversing the obstruction leads to a reduction in the process of renal tissue loss^14^. Based on this information and the need for an objective parameter for comparisons, we decided to measure the renal parenchyma thickness and compare animals submitted to obstruction and relief of UUO, of different ages and duration of obstruction. At first look, all animals subjected to obstruction presented a decrease in the parenchymal thickness in the obstructed kidney when compared with sham-operated rats, as expected. That shows an association between the renal damage caused by ureteral obstruction and loss of renal parenchyma.

It is also known that rats that recently finished nephrogenesis suffer more damage in renal development than older rats with complete mature nephrogenesis^15^, and that UUO leads to slowing maturation of interstitial, vascular, glomerular, and tubular cells^16^. Therefore, UUO must have a greater impact on kidneys with ongoing nephrogenesis or recent end of this process. These data can justify our findings in the present study when comparing groups of different ages using the correction method available (the mean thickness of sham-operated rats), which showed statistically significant results of older rats preserving more renal parenchyma than newborn rats (both some NB *versus* Y and NB *versus* A groups). These data may also justify the findings obtained from the qualitative analysis showing that the obstructed kidneys from newborns had more severe damage after obstruction than older rats, with important parenchymal loss and tubular atrophy, but also, at the same time, the fraction of parenchyma that was preserved after obstruction relief had an overall healthier aspect (glomeruli and tubules) than in adults. This finding can be correlated to the capacity of kidney function preservation in newborns after relief of UUO. The damage caused by persistent obstruction, such as tubular atrophy, permanent activation of the renin-angiotensin-aldosterone system (that contributes to progressive interstitial fibrosis)^14^, and collagen deposition is attenuated after the relief, but it is not completely reversed^16^.

Considering that even after the reversion of the obstructive stress the mechanism of parenchymal damage persists, we may explain why our comparisons between groups subjected only to obstruction, and groups of the same age and period of obstruction that were relieved after two weeks presented no significant differences in renal parenchyma thickness at any age. This is a limitation that we recognize in the present study: the period of release of UUO before euthanasia and analysis was arbitrary. Probably, if we had followed for a longer period of observation, both renal function and histology, we would observe a better scenario of renal recovery after the pyelostomy.

The known data about the response of the non-obstructed kidney to obstructive processes is that the compensatory response is best observed in complete obstruction lesions, with weight gain in the contralateral kidney^17^. It is also known that compensatory hypertrophy is more significant in younger organisms, as it was demonstrated in a previous study in dogs submitted to experimental renal damage^13^, mainly in newborn animals^14^. Our study showed similar results: comparing different age groups using the correction method (by the mean renal thickness of respective sham rats), the renal parenchyma thickness of the contralateral kidney was statistically higher at younger ages, showing that the younger the age of obstruction, the better the compensation of the contralateral kidney, since renal hypertrophy leads to a gain in function. Our study had statistically significant results in comparisons between groups obstructed for four weeks. Such results in groups obstructed for a longer period collaborate with the current data from the literature on compensation of a healthy kidney after a loss of function or parenchyma in the other kidney.

Also, when comparing groups that were submitted to relief of UUO with UUO only, both in newborn and young groups, the current study showed that animals that were not submitted to pyelostomy had thicker contralateral renal parenchyma than groups of the same age that were submitted to the relief surgery. This finding is compatible with literature data of attenuation in the compensation process after the reversion of the obstructive lesion18. Probably, hormonal mechanisms are involved in these responses, and this will be the objective of the next investigation utilizing the current model.

### Protein loss and renal function

Since renal protein loss and cellular transformation are all progressive during UUO, protein loss is expected to increase progressively during the evolution of obstruction. Comparing a one-week obstruction and a three-week obstruction in rats, it is known that a longer duration of obstruction results in a higher protein loss and worsening of renal function^10^.

In the present study, after obstruction all rats showed an increase in the albumin loss, and albuminuria was progressively higher in rats obstructed for longer periods. It was observed that the newborn rats presented a reduced Alb loss in comparison with the young and adult age groups when obstructed for four weeks, although no differences were verified in rats obstructed for two weeks. In addition, in the relief groups we verified that younger animals tended to present a higher Alb excretion, and this can be justified by the progression of glomerular injury that occurred through the different age groups. The newborns’ glomeruli were more preserved in the obstructed groups, which led to a higher protein loss when the animals were reoperated. The younger glomeruli tended to appear more atrophic, and the adult ones were mostly fibrosed. That is why they showed progressively lower protein loss after relief of obstruction when compared with younger animals.

Similar results were seen in the TP excretion from the rats of this study. The newborn age group showed lots of statistical differences. All groups presented higher protein loss when compared with their sham groups. Apparently, there is no progression in TP loss from newborns considering a two-week obstruction and a four-week obstruction. Even after relief, there is no difference between both durations of obstruction. There is also an important worsening in protein loss similar to Alb after relief in both groups, probably due to progressive glomerular damage that occurs in the age groups. Since the newborns tended to show healthier glomeruli, they tended to present greater protein loss. In the young and adult age groups, there were no significant differences, probably due to the same reasons previously discussed in the Alb section. Also, like albuminuria, TP loss is progressively worse for older rats in longer periods of obstruction when comparing different age groups.

Complete UUO initiates a rapid sequence of events in the obstructed kidney, leading in 24 hours to reduced renal blood flow and glomerular filtration rate^19^. This is followed within several days by hydronephrosis, interstitial macrophages infiltration, and tubular cell death attributable to apoptosis and necrosis. Tubular epithelial cell death is caused by several stressing agents resulting from UUO, including ischemia, hypoxemia, oxidant injury, and axial strain caused by tubular dilatation^20^. All those changes result in a hydronephrotic kidney unable to completely perform its function. In the present study, the worsening of renal function was measured by the capacity of the obstructed kidney to maintain its Cr and U excretion.

We verified that Cr excretion was worsened in most of the rats after ureteral obstruction. However, the animals of YR4, AR2, and AR4 did not present a difference in Cr excretion in comparison with the sham animals. Nonetheless, a significant percentage of the animal kidneys in these groups were not even able to produce U after pyelostomy. We verified that in older animal groups the creatinuria tended to decrease for longer periods of obstruction in comparison with the younger animals, probably due to the increased capacity for recuperation of renal function after the relief of obstruction. In young and adult rats, most of the groups presented a similar tendency for the differences shown in newborn rats, with lower Cr excretion after obstruction and even worse after the relief. Finally, in the older rats there was a tendency towards decreased capacity of Cr excretion in longer periods of obstruction.

Another verification of the current study was that U excretion was also worsened after an obstructive injury, in comparison with the sham groups. In newborn rats, there was no statistical difference in comparison to a two-week or a four-week obstruction, before and after the relief of obstruction, but there was a tendency to decreased U excretion in unobstructed rats. Young and adult rats tended to not produce urine after relief of obstruction and that worsened for longer periods of obstruction and for older rats. When comparing the age groups, we verified no significant difference between newborns and young rats, but in adult rats, the U excretion was decreased in comparison with the younger groups. This is a clear indication that in older rats obstructive injury is more harmful than in younger ones. We may conclude that the newborn rats can withstand a longer period of obstruction without having the same loss after the relief of obstruction in comparison with the young rats. Since the adult rats showed us the worse handling for an obstructive injury by not producing urine after relief of obstruction and by showing a worse renal function during different obstruction periods, it is possible that younger rats that have not already gone through the glomeruli maturation process present better handling of these obstructive injuries than older rats.

## Conclusion

In the present study, we developed an experimental model to test UUO and its repercussions in different durations of obstruction and age groups.

The study of the renal function of obstructed rats showed us that a shorter obstruction leads to a better prognosis. Also, younger rats tended to show a better recovery phase after relief of obstruction than older rats, and this fact may be justified by the fact that histologically the younger rats tended to present a more extensive destruction of renal parenchyma, but in the remaining apparently normal tissue, the renal parenchyma of younger rats looked healthier than the parenchyma of older rats.

Previous knowledge in the literature about obstructive injuries reinforces the findings in our study that obstructive lesions are more harmful to a newly formed kidney and that the earlier the reversion of the obstruction, the better are the recovery and preservation of function in the organ. It also reinforces that contralateral kidney compensatory growth is proportional to the duration of UUO and tends to slow down, but it does not cease after relief. Lastly, it reinforces our findings that the younger the rat, the better is the compensatory growth of the contralateral kidney.

To better understand if the lack of urine production in older rats is due to a worse UUO recovery potential or if it is due to another factor, and to better clarify the histological process in relief of obstruction, further studies could increase the number of rats in each group and the follow up after relief of obstruction.

## Data Availability

All dataset were generated or analyzed in the current study.
